# Changes in family structure and children’s screen time: a longitudinal study of Australian children

**DOI:** 10.1007/s00431-026-07569-z

**Published:** 2026-11-09

**Authors:** Nigusie Shifera, Rebecca Pagano, Taren Sanders

**Affiliations:** 1 Institute for Positive Psychology and Education, Australian Catholic University, North Sydney, NSW Australia; 2 School of Public Health, Mizan Tepi University, Mizan Aman, Ethiopia; 3 National School of Education, Australian Catholic University, Strathfield, NSW Australia

**Keywords:** Screen time, Children, Family structure, Family transitions, Screen use

## Abstract

Children’s screen time varies by family structure, but evidence remains cross-sectional, making it difficult to determine whether differences reflect family structure or pre-existing sociodemographic inequalities. To our knowledge, no study has used matched comparison groups to test whether screen time changes following family transitions (two-parent to single-parent and vice versa). Using data from the Longitudinal Study of Australian Children, a nationally representative study drawing on the Baby and Kindergarten cohorts to follow children aged 6 to 13 (*N* = 8706 at baseline; mean age, 6.86 years; 51% male; 85% in two-parent households), we used pre-registered multilevel mixed-effects models to examine associations between family structure, sibling number, and screen time, and propensity score-matched piecewise regression to test changes in screen time following family transitions. After full adjustment, living in a single-parent household was not significantly associated with screen time (*B* = 11.98 min/week; 95% CI, − 7.41 to 31.37). Transitions from two-parent to single-parent (*B* = 18.45 min/week; 95% CI, − 94.48 to 131.39) or from single-parent to two-parent (*B* = − 49.47 min/week; 95% CI, − 247.58 to 148.64) were not associated with changes in screen time. In contrast, each additional sibling was associated with lower screen time (*B* = − 22.71 min/week; 95% CI, − 30.18 to − 15.25).

*Conclusion*: The association between family structure and screen time is largely explained by sociodemographic factors, and family transitions do not produce significant changes in screen time. Interventions to reduce screen time may be better directed at sociodemographic inequalities rather than at family structure or transitions, while considering siblings.

**Table:** 

**What is Known:** • *Most evidence on family structure and children's screen time is cross-sectional, leaving unclear whether family structure or pre-existing family differences explain observed patterns.* • *No study has used matched comparison groups to examine whether children's screen time trajectories shift following a family structure transition.*
**What is New:** • *Screen time levels and trajectories were compared before and after transitions in both directions, between children who transitioned and matched children in stable households.* • *After adjustment, family structure was not associated with screen time, and transitions were not associated with changes in screen time; having more siblings was consistently associated with lower screen time.*

**Supplementary Information:**

The online version contains supplementary material available at https://doi.org/10.1007/s00431-026-07569-z.

## Introduction

In recent decades, the landscape of digital media use among children has shifted, with initial exposure declining from approximately age 4 in the 1970s to as early as 4 months today [1]. In Australia, excessive screen time has become a primary concern for parents. Only 17% to 23% of preschoolers and 15% of school-aged children meet the recommended daily limits of less than 1 h and 2 h, respectively [2].

Children’s digital engagement is influenced by the broader household environment, including family structure, composition, and transitions [3]. Family structure refers to the number and types of parents or guardians residing in the child’s home (e.g., biological, adoptive, or foster) and whether the child lives in a single- or two-parent household. Household composition describes other resident members, such as the number of siblings [4], whereas family transitions refer to changes in family structure over time, either additive (e.g., moving from a single- to a two-parent household) or subtractive (e.g., moving from a two- to a single-parent household) [5].

Research on the effects of family structure and number of siblings on children’s screen time has produced inconsistent findings, with some studies reporting higher odds of excessive screen time among children in single-parent households [6], possibly due to fewer joint mediations [7], while having more siblings has been associated with lower screen time [8, 9], perhaps because siblings serve as alternative play partners [10], and shared resources also limit children’s screen access [11]. However, other studies find no meaningful associations with either family structure [12, 13] or sibling number [14]. These inconsistencies probably result from methodological limitations, as most studies use cross-sectional designs that cannot separate the effects of family structure from pre-existing family differences. For example, if families who later separate already differ in screen use before separation, a cross-sectional association with family structure could be spurious.

In addition to family structures and composition, transitions can play a critical role in shaping children’s screen time [15]. A transition to a single-parent household requires additional time and energy to manage logistics and work, leading to decreased parental engagement and supervision, which, in turn, may increase children’s screen time [16]. The emotional stress of family separation may lead parents to adopt a permissive approach to screen time [17]. Children may also experience significant emotional distress from family separation and turn to screens as a coping mechanism for feelings of loneliness, confusion, or sadness [18, 19].

Conversely, a transition from single-parent to two-parent households may reduce children’s screen time by expanding supervisory capacity, alternative activities, and structured engagement [20]. However, no study has examined whether family transitions are followed by inflection points in children’s screen time, a critical turning point at which the trajectory of screen time measurably shifts following a change in family structure.

Therefore, this study aimed to: (i) examine the associations between family structure and the number of siblings with children’s screen time; and (ii) assess whether family transitions (two- to single-parent and vice versa) produce inflection points in children’s screen time trajectories. The findings have implications for policymakers and practitioners: understanding how family structure and the number of siblings influence screen time can guide family-based interventions, and identifying inflection points following household transitions can inform the timing and targeting of support services.

## Methods

### Protocol registration

This study was prospectively registered on OSF (https://doi.org/10.17605/OSF.IO/U3JB4) and conducted in accordance with the STROBE reporting guidelines [21].

### Study design and participants

Data were drawn from the Longitudinal Study of Australian Children (LSAC; “Growing Up in Australia”), a nationally representative study designed to examine how early-life experiences shape developmental outcomes from middle childhood into late adolescence. The study began in 2004 and included two cohorts, Baby (0–1 years) and Kindergarten (4–5 years), each comprising approximately 5000 children, with data collected every 2 years [22, 23]. For the present study, we used data from both cohorts: the B (Baby) cohort (waves 4–7, 2010–2016) and the K (Kindergarten) cohort (waves 2–5, 2006–2012). Because the K cohort is 4 years older than the B cohort, these waves cover the same ages in both cohorts, approximately 6–7 to 12–13 years. Data from the two cohorts were combined and aligned by child age (Supplementary Table S1). The Australian Institute of Family Studies Ethics Committee approved the LSAC, and all participants provided written informed consent.

In total, 9043 children (32,392 observations) participated in at least one wave between ages 6–7 and 12–13. The analytic sample (i.e., children with at least two waves of valid screen time data) comprised 30,674 exposure-outcome pairs from 8471 children, with each pair linking family structure and screen time measured at the same wave (Table 1).

**Table 1:** Definition of study samples

Sample	Children	Observations	Definition
Full sample	9043	32,392	Children participating in at least one wave between ages 6–7 and 12–13
Baseline sample	8706	8706 (one per child)	Children participating at age 6–7; used to describe baseline characteristics (Table 2)
Analytic sample	8471	30,674	Children with at least two waves of valid screen time data

### Variables

#### Outcome

*Children’s screen time* was assessed via parent reports across three domains: television viewing, video game use, and computer/other electronic media use. Weekly screen time (minutes/week) was reported for each domain, and total screen time was calculated as the sum of all the three components.

#### Exposure variable

*Family structure* was assessed by identifying the parental figures residing in the home, including biological, adoptive, step, and foster parents responsible for caregiving. Households were classified as two-parent (two or more parents present) or single-parent (one parental figure present).

*Family transition* was defined as a change in family structure, including transitions from single-parent to two-parent families and vice versa. It was tracked by comparing family-type variables across consecutive waves. Children who experienced more than one transition (*n* = 128) were excluded from the transition analyses because they could not be assigned to a single transition direction.

*The number of siblings* was the count of siblings living in the household.

### Covariates

*Mother’s education* was defined as the highest level completed by the mother, as reported by the primary caregiver, and served as a proxy for socioeconomic position. Socioeconomic position (SEP) scores were not used because their construction is structurally dependent on household type, comprising three components for single-parent families (income, P1 education, P1 occupation) and five for two-parent families (adding P2 education and occupation) [24], meaning that single-parent households systematically score lower, and simultaneous adjustment could attenuate the exposure effect. Socio-Economic Indexes for Areas (SEIFA) are derived by the Australian Bureau of Statistics [25], from census data on income, education, employment, occupation, housing, and family structure to capture area-level disadvantage [3]. Additional covariates include child age (years); sex (male, female); Indigenous status (Indigenous, non-Indigenous); and home language (English, not English).

### Data processing

Screen time variables were examined for outliers using the interquartile range (IQR) method. Values exceeding Q3 + 1.5 × IQR were flagged as outliers. Outlier values were set to missing before imputation. Distributional improvements were assessed by comparing skewness before and after outlier treatment (Supplementary Table S2).

### Missing data handling

Missing data were addressed using multiple imputations via the mice package [26]. A tailored strategy was applied to the K cohort wave 2, where screen time was recorded as daily values: when both weekday and weekend values were present, weekly totals were derived using the formula (5 × weekday + 2 × weekend); when one value was missing, it was imputed before deriving the weekly total; when both weekday and weekend values were missing, the weekly total for that screen time type could not be computed and was treated as missing. Wave-level exclusions were applied when two or more screen time components (television, video games, computer/other electronic media) were missing, and children with fewer than two valid waves were excluded.

The imputation model accounted for the nested data structure (repeated measures within children) using predictive mean matching [27, 28]. The model included screen time outcomes and demographic covariates (family structure, number of siblings, age, sex, home language, mother’s education, SEIFA, Indigenous status), with the child identifier as the clustering variable to account for repeated measures. Twenty imputed datasets were generated, with 10 iterations per dataset. Imputations were performed separately for each cohort and then combined post-imputation. Density plots comparing the distributions of observed and imputed values confirmed that the imputed values were plausible and consistent with the observed data for both cohorts (Supplementary Fig. S1).

### Statistical analysis

#### Longitudinal data structure

Screen time was analyzed as a repeated outcome, with observations nested within children. Because family structure and screen time both change during childhood, linking them at each wave captures changes that a single baseline-to-outcome comparison would miss. All waves with valid data, including intermediate waves, were included, so each child contributed up to four observations. Baseline was the first wave with valid data: age 6–7 (wave 4, B cohort; wave 2, K cohort) for 96.3% of children (8706 of 9043) and a later wave for the remaining 337 (3.7%; Supplementary Table S1). Family structure and number of siblings were linked to screen time measured at the same wave; number of siblings was an exposure in the household composition analysis and a time-varying covariate in the transition analysis. Time-invariant covariates (sex, Indigenous status, home language, and maternal education) were taken from the first wave with available data, and time-varying covariates (age and SEIFA) were taken from the same wave as the outcome.

#### Household composition analysis

We used a multilevel mixed-effects model to examine the relationships between family structure, number of siblings, and children’s screen time, accounting for the longitudinal structure of the data, with observations nested within children, who were nested within cohorts. The models evaluated whether screen time differed significantly by family structure and number of siblings, while controlling for age, sex, mother’s education, SEIFA, Indigenous status, and home language. Residual diagnostics showed approximate normality, with mild departures in the tails, consistent with the bounded nature of screen time data (Supplementary Fig. S2).

#### Family transition analysis

To assess whether children’s screen time shifted at the point of family structure change, we estimated piecewise linear mixed-effects models separately for (i) single-parent to two-parent transitions and (ii) two-parent to single-parent transitions. For each child experiencing a transition, the transition wave was identified. Time was recentered relative to this transition point (e.g., if the transition occurred at wave 5, waves 4–7 were coded − 1, 0, + 1, + 2). Three piecewise terms were constructed: (i) a pre-transition time variable capturing the slope before the transition, (ii) a post-transition indicator (0 = before; 1 = at or after transition) capturing an immediate level change, and (iii) a post-transition time variable capturing the slope after the transition.

Children experiencing household transitions may systematically differ from those in stable households [29]. To improve comparability between children who did and did not experience a change in family structure, we used propensity score matching [30, 31]. A propensity score is a child’s estimated probability of experiencing a transition, based on age, sex, mother’s education, SEIFA, Indigenous status, number of siblings, home language, and screen time, all measured at baseline before the transition. Each child who experienced a transition was paired with a child from a stable household with the most similar score (1:1 nearest-neighbor matching), with each stable child used only once (without replacement) and only if the scores differed by less than 0.25 standard deviations (caliper). For single- to two-parent transitions, transitioned children were matched with those in stable single-parent households; for two- to single-parent transitions, transitioned children were matched with those in stable two-parent households. Covariate balance was assessed using standardized mean differences; after matching, all covariates fell within the ± 0.1 threshold, confirming adequate balance for both transition directions (Supplementary Fig. S3).

After matching, three-level piecewise linear mixed-effects models were fitted, with repeated observations nested within children and children nested within cohorts. Time was centered on the transition point (or the pseudo-transition point for matched stable children, defined as the transition wave of their matched partner). Trajectories were modeled with a pre-transition slope, a level change at the transition, and a post-transition slope, including interactions between transition group and each piecewise component. Models were also adjusted for age, sex, mother’s education, SEIFA, Indigenous status, home language, and number of siblings. The primary parameter was the transition group × level-change interaction, which tested whether the immediate shift in screen time differed between children who transitioned and the stable groups.

### Sensitivity analysis

To test the robustness of our findings, we repeated the piecewise models without matching, including all transitioned and stable children and adjusting for the same covariates. Each stable child was assigned a random pseudo-transition wave from the same cohort.

## Results

### Sample characteristics

At baseline, demographic data were reported for 8706 participants (B cohort: *n* = 4242; K cohort: *n* = 4464). Both cohorts were comparable with respect to demographic characteristics: approximately 51% were male, the mean age was 6.86 years, and the majority were English-speaking (~ 90%) and Australian-born (99.6% in the B cohort and 95.9% in the K cohort). Most children lived in two-parent households (~ 85%), with biological mothers present in ~ 99% of households and biological fathers in ~ 83% (Table 2).

**Table 2:** Baseline sample characteristics (age 6–7 years)

Characteristics	B cohort	K cohort
Sample description		
Participant ***n*** (%)	4242 (48.7)	4464 (51.3)
Demographics		
Age (years), mean (SD)	6.85 (0.29)	6.86 (0.24)
Sex (male), ***n*** (%)	2187 (51.6)	2276 (51.0)
Indigenous, ***n*** (%)	145 (3.4)	153 (3.4)
English at home, ***n*** (%)	3843 (90.7)	3947 (88.4)
Born in Australia, ***n*** (%)	4227 (99.6)	4280 (95.9)
Household composition		
Biological mother, ***n*** (%)	4199 (99.0)	4393 (98.4)
Biological father, ***n*** (%)	3549 (83.7)	3677 (82.4)
Adoptive mother, ***n*** (%)	4 (0.1)	10 (0.2)
Adoptive father, ***n*** (%)	5 (0.1)	9 (0.2)
Stepmother, ***n*** (%)	5 (0.1)	10 (0.2)
Stepfather, ***n*** (%)	106 (2.5)	113 (2.5)
Foster mother, ***n*** (%)	1 (0.0)	1 (0.0)
Foster father, ***n*** (%)	1 (0.0)	1 (0.0)
Unknown mother, ***n*** (%)	1 (0.0)	1 (0.0)
Unknown father, ***n*** (%)	1 (0.0)	6 (0.1)
Two-parent household, ***n*** (%)	3636 (85.8)	3771 (84.7)
Parent count, mean (SD)	1.86 (0.35)	1.85 (0.36)
Single-parent household, ***n*** (%)	600 (14.2)	679 (15.3)
Number of siblings, mean (SD)	1.59 (1.01)	1.58 (1.04)
Screen time (min/week)		
TV, mean (SD)	770.43 (381.54)	797.82 (380.91)
Video games, mean (SD)	279.33 (251.70)	195.79 (231.20)
Computer/other electronic media, mean (SD)	210.57 (232.05)	250.99 (223.94)
Total, mean (SD)	1236.99 (555.99)	1215.65 (561.97)

*B cohort* Baby cohort, *K cohort* Kindergarten cohort

### Screen time by household characteristics

Descriptive statistics showed that children in single-parent households reported higher total screen time than those in two-parent households (1301 vs. 1210 min/week) (Supplementary Table S3). These differences were consistent across age, cohort, and sex (Supplementary Fig. S4). Screen time decreased with the number of siblings, from 1278 min/week among only children to 1209 min/week among those with three or more siblings (Supplementary Table S3). These patterns were consistent across age, cohort, and sex (Supplementary Fig. S5).

### Associations between family structure, siblings, and screen time

In unadjusted analyses, children in single-parent households had higher screen time than those in two-parent households (*B* = 89.80 min/week, 95% CI: 69.28 to 110.32, *p* < 0.001). After adjusting for sociodemographic covariates, the association was substantially attenuated and no longer statistically significant (*B* = 11.98, 95% CI: − 7.41 to 31.37, *p* = 0.226), reflecting an 87% reduction from the unadjusted estimate (Table 3, Panel A). The association did not vary by child sex (pooled likelihood ratio test, D1 = 0.283, *p* = 0.595; Supplementary Table S4).

**Table 3:** Associations of family structure and number of siblings with children’s weekly screen time

Model	*B*	95% CI	*P*
**Panel A: Single-parent household (reference: two-parent)**			
Model 1: Unadjusted	89.801	69.280, 110.323	< 0.001
Model 2: + Age, sex	36.582	17.060, 56.104	< 0.001
Model 3: + Mother’s education	16.705	− 2.632, 36.043	0.090
Model 4: + SEIFA, Indigenous, language	11.983	− 7.405, 31.370	0.226
**Panel B: Number of siblings (per additional sibling)**			
Model 1: Unadjusted	− 12.267	− 20.074, − 4.459	0.002
Model 2: + Age, sex	− 13.953	− 21.552, − 6.355	< 0.001
Model 3: + Mother’s education	− 21.197	− 28.646, − 13.748	< 0.001
Model 4: + SEIFA, Indigenous, language	− 22.714	− 30.176, − 15.251	< 0.001
**Panel C: Mutually adjusted**			
Single-parent household (ref: two-parent)	5.323	− 14.174, 24.820	0.593
Number of siblings	− 22.469	− 29.983, − 14.955	< 0.001
Age	82.699	80.023, 85.375	< 0.001
Sex: female	− 113.231	− 129.759, − 96.703	< 0.001
Mother education: postgraduate	− 242.782	− 276.952, − 208.611	< 0.001
Mother education: graduate diploma	− 168.878	− 203.086, − 134.669	< 0.001
Mother education: bachelor’s degree	− 193.144	− 218.493, − 167.794	< 0.001
Mother education: advanced diploma	− 97.795	− 129.209, − 66.382	< 0.001
Mother education: certificate/trade	− 19.551	− 41.907, 2.805	0.072
Mother education: other qualification	− 59.557	− 124.290, 5.175	0.071
SEIFA z-score	− 25.466	− 33.311, − 17.620	< 0.001
Indigenous	23.956	− 26.893, 74.806	0.356
Language: non-English	− 25.926	− 54.157, 2.304	0.072

*B *unstandardized coefficient (minutes/week), *CI* confidence interval*. *Panel A: Reference category is two-parent household. Panel B: continuous siblings count. Models 1–4 show sequential covariate adjustment. Panel C: Mutually adjusted model with both predictors and all covariates. Reference categories: two-parent household, male, non-Indigenous, English-speaking. Mother’s education reference: year 12 or below. Age, number of siblings, and SEIFA z-score are centered.

The association between the number of siblings and screen time remained robust across all model specifications. In unadjusted analyses, each additional sibling was associated with 12.27 fewer minutes of weekly screen time (95% CI: − 20.07 to − 4.46; *p* = 0.002). After adjusting for covariates, the association strengthened (*B* = − 22.71, 95% CI: − 30.18 to − 15.25, *p* < 0.001) (Table 3, Panel B). The association did not differ by child sex (D1 = 2.192, *p* = 0.140; Supplementary Table S4).

In a mutually adjusted model, both family structure and number of siblings were included; the pattern of results remained consistent. Family structure was not significantly associated with screen time (*B* = 5.32 min/week, 95% CI: − 14.17 to 24.82, *p* = 0.593), providing no evidence of a difference by family structure after accounting for the number of siblings. In contrast, the protective association between siblings and screen time remained significant and was independent of family structure (*B* = − 22.47 min/week, 95% CI: − 29.98 to − 14.96, *p* < 0.001) (Table 3, Panel C).

### Family structure transition and screen time

For transition analyses, the analytic sample comprised 8471 children classified into five groups: stable two-parent households (*n* = 6469), stable single-parent households (*n* = 895), two-to-single transitions (*n* = 701), single-to-two-parent transitions (*n* = 278), and multiple transitions (*n* = 128). Propensity score matching achieved high retention: 100% of two-to-single transitions (701 pairs) and 100% of single-to-two transitions (278 pairs) were matched to comparable children from stable households.

### Single-to two-parent transition

**Fig. 1:**
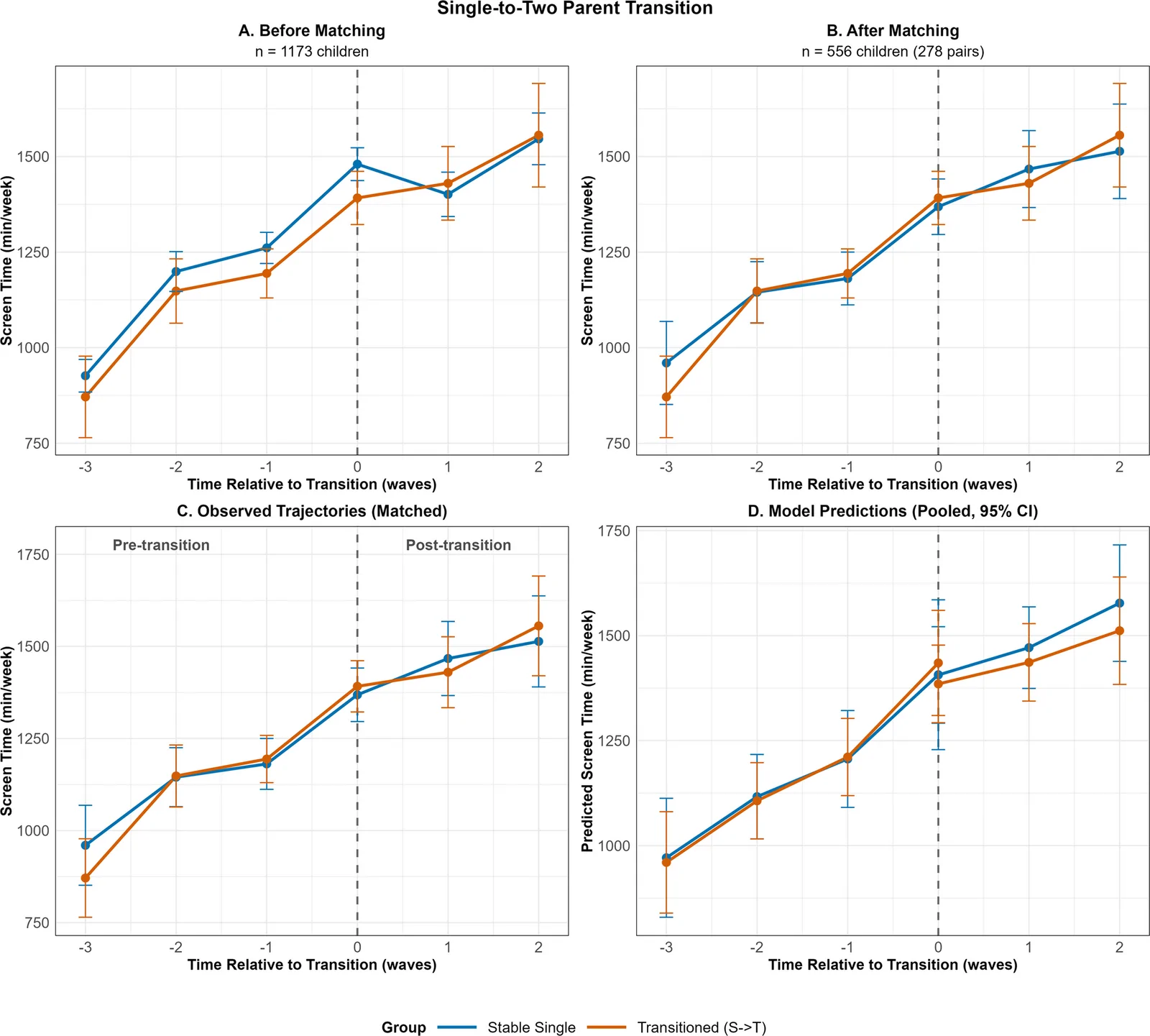
Screen time trajectories for children experiencing single-to-two-parent transitions compared to matched children from stable single-parent households. **A** Raw trajectories before matching. **B** Trajectories after propensity score matching. **C** Observed matched trajectories with pre- and post-transition periods indicated. **D** Model-predicted trajectories

After propensity score matching, 278 children who transitioned from single-parent to two-parent households were compared with matched children from stable single-parent households. The differential level change at the transition did not differ significantly between groups (*B* = − 49.47, 95% CI: − 247.58 to 148.64, *p* = 0.622). Neither the pre-transition slope difference (*B* = 2.88, 95% CI: − 95.89 to 101.66, *p* = 0.954) nor the post-transition slope difference (*B* = − 13.99, 95% CI: − 118.57 to 90.59, *p* = 0.792) differed significantly between groups, indicating parallel trajectories throughout the observation period. Both groups showed increases in screen time, with the stable group demonstrating slopes of 55.54 min/week per wave pre-transition (95% CI: − 30.27 to 141.35; *p* = 0.202) and − 4.93 min/week per wave post-transition (95% CI: − 93.47 to 83.62; *p* = 0.912; Table 4, Panel A; Fig. 1).

**Table 4:** Piecewise regression model estimates of screen time (minutes/week) before and after family structure transitions

Parameter	*B*	95% CI	*p*
**Panel A: Single-to-two-parent transition (*n* = 278 matched pairs)**			
* Stable group trajectory (reference)*			
Intercept (stable group)	546.1	251.3, 840.9	< 0.001
Slope before transition	55.5	− 30.3, 141.4	0.202
Level change at transition	− 0.5	− 171.3, 170.3	0.995
Slope after transition	− 4.9	− 93.5, 83.6	0.912
* Difference: transition group vs stable group*			
Pre-transition group difference	3.2	− 192.1, 198.5	0.974
Difference in slope before transition	2.9	− 95.9, 101.7	0.954
** Difference in level change at transition†**	− 49.5	− 247.6, 148.6	0.622
Difference in slope after transition	− 14.0	− 118.6, 90.6	0.792
**Panel B: Two-to-single-parent transition (*n* = 701 matched pairs)**			
* Stable group trajectory (reference)*			
Intercept (stable group)	610.9	386.8, 835.0	< 0.001
Slope before transition	34.5	− 18.0, 87.0	0.196
Level change at transition	− 21.7	− 112.3, 68.9	0.636
Slope after transition	16.9	− 37.5, 71.3	0.539
* Difference: transition group vs stable group*			
Pre-transition group difference	24.0	− 84.1, 132.1	0.662
Difference in slope before transition	8.3	− 50.8, 67.3	0.783
** Difference in level change at transition†**	18.5	− 94.5, 131.4	0.748
Difference in slope after transition	− 47.5	− 111.3, 16.4	0.144

*B *unstandardised coefficient (minutes/week), *CI *confidence interval. Time is centered at the transition wave (pseudo-transition wave for matched stable children). Slopes are the change per wave; level change is the shift at the transition wave. Difference parameters compare the transition group with the stable group; positive values indicate higher screen time or a more positive slope. †Primary parameter: tests whether the shift in screen time at the transition wave differed between groups. Panel A: stable group = stable single-parent households; Panel B: stable group = stable two-parent households. Models were adjusted for age, sex, mother’s education, SEIFA, Indigenous status, home language, and number of siblings; estimates were pooled across 20 imputed datasets

### Two-to single-parent transition

**Fig. 2:**
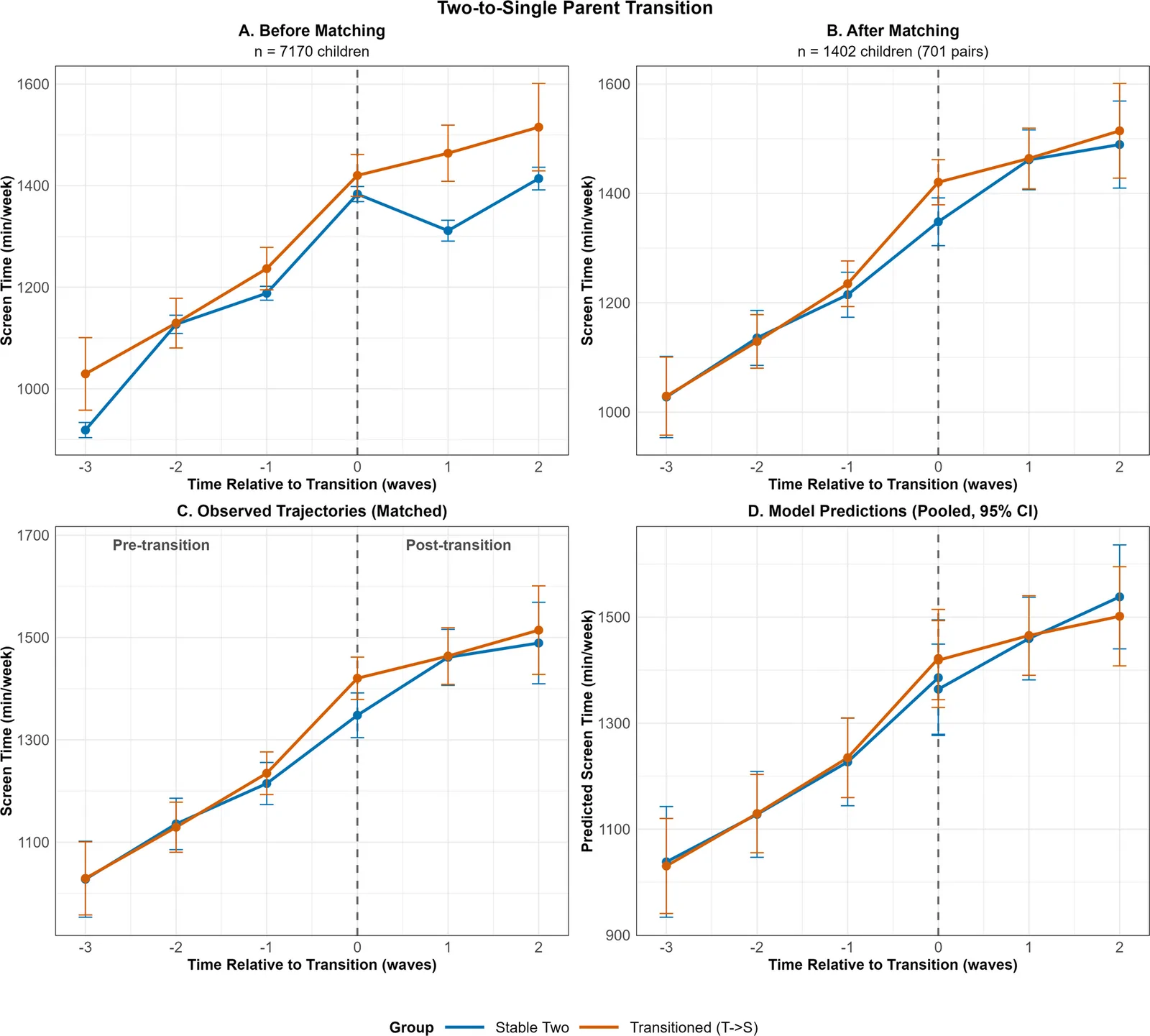
Screen time trajectories for children experiencing two-to-single-parent transitions compared with matched children from stable two-parent households. **A** Raw trajectories before matching. **B** Trajectories after propensity score matching. **C** Observed matched trajectories with pre- and post-transition periods indicated. **D** Model-predicted trajectories

After propensity score matching, 701 children who transitioned from two-parent to single-parent households were compared with matched children from stable two-parent households. The primary parameter of interest, the differential level change at transition, did not differ significantly between groups (*B* = 18.45, 95% CI: − 94.48 to 131.39, *p* = 0.748). Neither the pre-transition slope difference (*B* = 8.26, 95% CI: − 50.83 to 67.34, *p* = 0.783) nor the post-transition slope difference (*B* = − 47.46, 95% CI: − 111.30 to 16.38, *p* = 0.144) differed between groups, indicating parallel trajectories throughout the observation period. Both groups showed increases in screen time, with the stable group demonstrating slopes of 34.49 min/week per wave pre-transition (95% CI: − 17.99 to 86.97; *p* = 0.196) and 16.92 min/week per wave post-transition (95% CI: − 37.46 to 71.30; *p* = 0.539; Table 4, Panel B; Fig. 2).

### Sensitivity analysis

In the unmatched analysis including all transitioned and stable children, the level change at the transition remained non-significant for both single-to-two-parent (*B* = − 62.8, 95% CI: − 198.9 to 73.3, *p* = 0.366) and two-to-single-parent transitions (*B* = − 11.8, 95% CI: − 86.5 to 62.9, *p* = 0.756). Before a two-to-single-parent transition, children who later transitioned had higher screen time than those in stable two-parent households (*B* = 75.1, 95% CI: 3.6 to 146.5, *p* = 0.039); this difference was attenuated after matching (*B* = 24.0, 95% CI: − 84.1 to 132.1, *p* = 0.662). The post-transition slope for two-to-single-parent transitions was lower among transitioned children in the unmatched analysis (*B* = − 58.4, 95% CI: − 97.5 to − 19.3, *p* = 0.003), with a similar but less precise estimate in the matched analysis (*B* = − 47.5, 95% CI: − 111.3 to 16.4, *p* = 0.144) (Supplementary Table S5).

## Discussion

The association between family structure and children’s screen time was no longer significant after adjustment for sociodemographic factors, suggesting that observed differences between single- and two-parent households are largely attributable to sociodemographic disparities rather than household structure itself. This finding aligns with a nationally representative Canadian study that found no meaningful association between family structure and youth screen time [12]. However, other studies have reported that family structure is significantly associated with screen time [6, 32]; a discrepancy may reflect differences in sociodemographic adjustment across studies. Without adequate control for these factors, the effects of sociodemographic disadvantage may be incorrectly attributed to family structure [33]. For instance, single-parent households typically have lower incomes and fewer resources, which are independently associated with higher screen time [34]. This suggests that family structure alone may be a limited indicator of children at risk of excessive screen time. Interventions to reduce children’s screen time may therefore be better directed at sociodemographic inequalities, such as differences in maternal education and area-level disadvantage, rather than at family structure alone.

The number of siblings was inversely associated with children’s screen time, with each additional sibling corresponding to about 23 fewer minutes of screen time per week. Notably, the association slightly strengthened after sociodemographic adjustment, suggesting that the protective effect of siblings is not explained by sociodemographic differences associated with having more siblings and may even be suppressed by them. This finding aligns with prior research showing that children with siblings engage in less screen-based activity than only children [32].

Several mechanisms may explain this association. Siblings serve as alternative play partners, reducing reliance on screen-based entertainment while promoting active, social play through peer modeling and co-participation in physical activities [10]. Shared attention and resources may naturally limit individual screen time and encourage cooperative, non-screen activities. Although the difference of approximately 23 min per sibling per week is small at the individual level, it may become meaningful when accumulated across siblings and over the course of childhood. For only children, encouraging interactive, socially engaged play may be more effective than simply restricting solitary screen use.

This study also examined whether family transitions were followed by an immediate change in children’s screen time. The level change at the transition point was not statistically significant for either the single-to-two-parent transition (*B* = − 49.47, *p* = 0.622) or the two-to-single-parent transition (*B* = 18.45, *p* = 0.748). In the matched analyses, the slopes before and after the transition did not differ significantly between the transition and stable groups. The absence of an immediate level change did not depend on matching, as it remained non-significant in a sensitivity analysis without matching. These findings do not support the family instability hypothesis, which suggests that family changes disrupt parenting routines and supervision, leading to worse child outcomes [29]. However, it aligns with a systematic review that concluded the instability hypothesis is inconclusive, with many studies reporting no association or associations limited to certain groups and transition types [35]. Similarly, no association was found between family instability and children’s behavioral outcomes among those born to cohabiting or single parents [36]. The current findings extend this finding by demonstrating that screen time, as a behavioral outcome, also remains unaffected by family transitions once pre-existing group differences are controlled for through matching. Screen time guidance may therefore be appropriate for all families, rather than only for families undergoing separation or re-partnering.

Our findings also differ from those of Cano and Gracia’s analysis of LSAC data [37], which reported that parental separation increased children’s unstructured screen-based activities using a fixed-effects model. Two methodological distinctions likely contribute to this discrepancy. First, our definition of family structure transition is broader, encompassing changes involving biological, adoptive, step, and foster parental figures, whereas Cano and Gracia’s analysis was restricted to two-biological-parent households. Second, the use of propensity score matching established counterfactual comparison groups matched with covariates [38], separates the transition effect from the selection processes that differentiate families who do or do not experience family structure change. The absence of a significant effect after matching suggests that differences in screen time across family types may reflect pre-existing differences between families rather than the transition event itself [39].

### Strengths and limitations

This study has several notable strengths. First, it uses longitudinal data from a nationally representative study (LSAC), enabling examination of within-child changes in screen time. Second, to our knowledge, it is the first study to examine whether family transitions produce distinct inflection points in children’s screen time trajectories. Third, the application of propensity score matching strengthened causal inference by comparing children who transitioned with peers who were observationally similar and in stable households, thereby reducing the risk of confounding by sociodemographic factors. Fourth, missing data were handled using multiple imputation, preserving the data’s distribution and accounting for nesting via a clustering variable, thereby reducing potential bias from non-random missingness.

Several limitations should be acknowledged. First, screen time was assessed via parent report, which is subject to recall and social desirability biases and may underestimate or overestimate children’s actual use [40, 41]. Future research should use frequent, objective measures of screen time to address these limitations. Second, the data were collected between 2006 and 2016 and might not fully represent current patterns of children’s screen use, including smartphone and streaming platform use. In addition, the two cohorts were observed at the same ages in different calendar periods, so secular changes in screen use over this period may have influenced the estimates. Nevertheless, replication in more recent cohorts that capture smartphone, social media, and streaming use is needed. Third, the 2-year intervals between LSAC waves mean that the precise timing of family structure transitions and any immediate screen-time response may not be captured accurately; shorter-term fluctuations between waves would be missed. Studies with more frequent measurements around the time of a transition are needed to capture these changes. Fourth, the data captured screen time quantity but not content or context, such as whether use was educational, passive, or involved co-viewing, limiting our ability to distinguish between beneficial and harmful forms of engagement. Future studies should therefore examine device- and content-specific screen time rather than total time alone.

## Conclusions

Family structure was not independently associated with screen time after adjusting for sociodemographic factors, and we found no evidence that transitions between two-parent and single-parent households were followed by changes in screen time. In contrast, having more siblings was associated with less screen time. These findings suggest that efforts to reduce screen time may be better directed at sociodemographic inequalities, such as differences in maternal education, rather than at family structure or its transitions alone, while also considering the role of siblings.

## Supplementary Information

Below is the link to the electronic supplementary material.

ESM 1 (DOCX 1.35 MB)

## Data Availability

The LSAC dataset is available from the National Centre for Longitudinal Data (https://growingupinaustralia.gov.au), subject to approval from the Australian Institute of Family Studies. The preregistered study protocol, including the analysis plan, is available on OSF (https://doi.org/10.17605/OSF.IO/U3JB4). The R code for this study is available at https://github.com/NiguseAylie/LSAC_Study.
