# Supplementary material for: Changes in family structure and children’s screen time: a longitudinal study of Australian children
Source: Eur J Pediatr. 2026 Nov 9;185(12):922. doi: 10.1007/s00431-026-07569-z (PMC13745404; doi:10.1007/s00431-026-07569-z)
Supplement: Supplementary file 1 [file 431_2026_7569_MOESM1_ESM.docx]

Table S1. Number of children participating at each wave, by cohort and child age

| **Child age (years)** | **B cohort wave (year)** | **B cohort n** | **K cohort wave (year)** | **K cohort n** | **Total n** |
| --- | --- | --- | --- | --- | --- |
| 6–7 | Wave 4 (2010) | 4,242 | Wave 2 (2006) | 4,464 | 8,706 |
| 8–9 | Wave 5 (2012) | 4,085 | Wave 3 (2008) | 4,331 | 8,416 |
| 10–11 | Wave 6 (2014) | 3,764 | Wave 4 (2010) | 4,169 | 7,933 |
| 12–13 | Wave 7 (2016) | 3,381 | Wave 5 (2012) | 3,956 | 7,337 |
| Total observations |  | 15,472 |  | 16,920 | 32,392 |
| Unique children |  | 4,392 |  | 4,651 | 9,043 |

*Note: B = Baby cohort; K = Kindergarten cohort. Counts are for the full sample (children participating in at least one wave between ages 6–7 and 12–13). The number of unique children exceeds the baseline sample (n = 8,706) because some children joined at a later wave.*

Table S2: Outlier Treatment and Distributional Improvement of Screen Time Variables

|  | **Skewness** | | **Outliers** | | **Mean (SD), min/week** | |
| --- | --- | --- | --- | --- | --- | --- |
| **Variable** | **Raw** | **Clean** | **n** | **%** | **Raw** | **Clean** |
| TV | 2.18 | 0.44 | 1,124 | 3.51 | 846 (514) | 785 (381) |
| Video games | 3.02 | 1.11 | 1,497 | 4.69 | 294 (380) | 235 (244) |
| Computer/Other | 3.75 | 1.08 | 1,560 | 5.40 | 303 (399) | 233 (228) |
| Total screen time | 2.32 | 0.49 | 1,060 | 3.71 | 1,431 (874) | 1,225 (560) |

***Note.*** *Outliers were identified using the interquartile range (IQR) method. Values exceeding Q3 + 1.5×IQR were flagged as outliers; extreme outliers exceeded Q3 + 3×IQR. Screen time is reported in minutes per week. N = total valid observations across all waves. Clean = after outlier removal.*

**Figure S1**: Diagnostic Density Plots Comparing Observed and Imputed Distributions

***Panel A.*** *B cohort (infant cohort). Density plots for continuous variables (screen time measures, SEIFA) and proportion plots for categorical variables (language at home, country of birth, maternal education). Red shading represents imputed values; blue shading represents observed values.*

***Panel B.*** *K cohort (kindergarten cohort). Density plots for continuous variables (screen time measures, SEIFA) and proportion plots for categorical variables (language at home, country of birth, maternal education). Red shading represents imputed values; blue shading represents observed values.*

Figure S2: Residual Diagnostics for the Nested Mixed-Effects Model.

***Panel A.*** *Normal Q–Q plot of residuals. Residuals followed the theoretical normal distribution closely through the centre of the distribution, with mild departures at the tails, indicating approximate normality consistent with the bounded nature of screen time data.*

***Panel B.*** *Residuals versus fitted values. The loss smoother (blue) remained close to the zero-reference line (red dashed), with a mild upward drift at higher fitted values. Given the large sample size (N = 30,674), these minor deviations do not threaten the validity of the model estimates.*

**Figure S3: *Covariate Balance for Propensity Score Matching.*** Standardised mean differences before (orange triangles) and after (blue circles) propensity score matching. Dashed vertical lines indicate the ±0.1 threshold for adequate balance.

**Panel A.** Single-to-two-parent transition. After matching, all covariates fell within the ±0.1 threshold, confirming adequate balance.

***Panel B.*** *Two-to-single-parent transition. After matching, nearly all covariates fell within or near the ±0.1 threshold, confirming adequate balance.*

Table S3**.** Screen Time (min/week) by Family Type and Number of Siblings

|  | N | TV | Video Games | Computer/Other | Total |
| --- | --- | --- | --- | --- | --- |
| Family Type | | | | | |
| Two-parent | 26,802 | 773 (377) | 231 (241) | 234 (226) | 1,210 (555) |
| Single parent | 5,414 | 842 (399) | 253 (262) | 230 (241) | 1,301 (575) |
| Difference |  | 69 | 22 | -4 | 91 |
| Number of Siblings | | | | | |
| Only child | 2,109 | 814 | 249 | 250 | 1,278 (590) |
| 1 sibling | 11,344 | 787 | 234 | 238 | 1,229 (559) |
| 2 siblings | 7,994 | 772 | 237 | 228 | 1,211 (557) |
| 3+ siblings | 3,825 | 790 | 225 | 223 | 1,209 (547) |

***Note****. Values are mean (SD) unless otherwise indicated. Difference = Single-parent minus Two-parent. TV, Video Games, and Computer/Other values are means only; Total includes SD*.

***Figure S4.*** *Screen time by household type across age, cohort, and sex. (A) Screen time components (TV, video games, computer/other) by household type and age. (B) Total screen time by cohort, age, and household type. (C) Total screen time by household type across age groups. (D) Total screen time by household type, age, and sex. Values are means with 95% confidence intervals. Screen time is measured in minutes per week.*

***Figure S5.*** *Screen time by number of siblings across age, cohort, and sex. (A) Screen time components (TV, video games, computer/other) by number of siblings and age. (B) Total screen time by cohort, age, and number of siblings. (C) Total screen time by number of siblings across age groups. (D) Total screen time by number of siblings, age, and sex. Values are means with 95% confidence intervals. Screen time is measured in minutes per week.*

Table S4**:** Interaction Tests (D1 Statistic)

| **Interaction** | **Mean Chi-Sq** | **df** | **D1 Statistic** | **P value** |
| --- | --- | --- | --- | --- |
| Family Structure × Siblings | 0.788 | 1 | 0.525 | 0.469 |
| Family Structure × Sex | 0.393 | 1 | 0.283 | 0.595 |
| Siblings × Sex | 3.001 | 1 | 2.192 | 0.140 |

Note: Interaction tests were conducted in the fully adjusted model using the D1 statistic for multiply imputed data. None of the interactions were statistically significant (p < 0.05).

**Table S5.** Piecewise mixed-effects model estimates of screen time (minutes/week) before and after family structure transitions: matched (primary) and unmatched (sensitivity) analyses

| **Parameter** | **Matched B (95% CI)** | **p** | **Unmatched B (95% CI)** | **p** |
| --- | --- | --- | --- | --- |
| **Panel A: Single-to-two-parent transition** | | | | |
| Pre-existing group difference | 3.2 (−192.1, 198.5) | 0.974 | 34.7 (−93.0, 162.4) | 0.594 |
| Difference in slope before transition | 2.9 (−95.9, 101.7) | 0.954 | 27.2 (−44.1, 98.5) | 0.454 |
| Difference in level change at transition† | −49.5 (−247.6, 148.6) | 0.622 | −62.8 (−198.9, 73.3) | 0.366 |
| Difference in slope after transition | −14.0 (−118.6, 90.6) | 0.792 | −29.4 (−106.9, 48.1) | 0.456 |
| **Panel B: Two-to-single-parent transition** | | | | |
| Pre-existing group difference | 24.0 (−84.1, 132.1) | 0.662 | 75.1 (3.6, 146.5) | 0.039 |
| Difference in slope before transition | 8.3 (−50.8, 67.3) | 0.783 | 7.6 (−31.0, 46.1) | 0.700 |
| Difference in level change at transition† | 18.5 (−94.5, 131.4) | 0.748 | −11.8 (−86.5, 62.9) | 0.756 |
| Difference in slope after transition | −47.5 (−111.3, 16.4) | 0.144 | −58.4 (−97.5, −19.3) | 0.003 |

*Note. B = unstandardised coefficient (minutes/week); CI = confidence interval. Parameters are defined as in Table 4; estimates are differences between the transition group and the stable group. †Primary parameter. Matched: 1:1 propensity score matching (Panel A: 278 matched pairs; Panel B: 701 matched pairs). Unmatched: all eligible children (Panel A: 278 transition, 895 stable; Panel B: 701 transition, 6,469 stable), with stable children assigned a random pseudo-transition wave from the same cohort. Both models were adjusted for the same covariates as in Table 4; estimates were pooled across 20 imputed datasets.*
